# Supplementary material for: Structural analysis of phosphoribosyltransferase-mediated cell wall precursor synthesis in Mycobacterium tuberculosis
Source: Nat Microbiol. 2024 Mar 15;9(4):976–87. doi: 10.1038/s41564-024-01643-8 (PMC10994848; doi:10.1038/s41564-024-01643-8)
Supplement: Supplementary file 1 — Supplementary Tables 1–3. [file 41564_2024_1643_MOESM1_ESM.pdf]

nature microbiology

Article

https://doi.org/10.1038/s41564-024-01643-8

Structural analysis of phosphoribosyltransferase-mediated cell wall precursor synthesis in *Mycobacterium tuberculosis*

In the format provided by the authors and unedited

Supplementary Table S1: Cryo-EM data collection, refinement and validation statistics

	**Rv3806c-DP** (EMDB-36072) (PDB 8J8K)	**Rv3806c-PRPP** (EMDB-36071) (PDB 8J8J)
**Data collection and processing**		
Magnification	130,000	130,000
Voltage(KV)	300	300
Electron exposure(e-/Å^2)	60	60
Defous range (μm)	-1.2 to -2.0	-1.2 to -2.0
Pixel size(Å)	0.96	0.96
Symmetry imposed	C3	C3
Initial particle images(no.)	3,228,225	3,597,451
Final particle images(no.)	272,777	410,532
Map resolution(Å)	3.36	2.76
FSC threshold	0.143	0.143
Map sharpening *B* factor(Å^2)	-135.7	-144.6
**Refinement**		
Initial model used(PDB code)	Alphafold2	8J8K
Model composition		
Non-hydrogen atoms	648,9	687,3
Protein residues	855	870
Ligands	3	9
Mean *B* factors(Å^2)		
Protein	74.87	51.47
Ligand	20.00	49.19
R.m.s.deviations		
Bond lengths(Å)	0.011	0.011
Bond angles(◦)	1.443	1.370
Validation		
MolProbity score	1.71	1.51
Clashscore	5.60	4.57
Poor rotamers(%)	0.00	0.00
Ramachandran plot		
Favored(%)	93.99	95.95
Allowed(%)	6.01	4.05
Disallowed(%)	0.00	0.00

Supplementary Table S2: Summary of model building

Protopmer Name	Chain	Total residues/ Range built	Poly-Ala model	Unmodelled residues	% atomic model	Ligands
**Rv3806c-DP**	A/B/C	302/18-302	79,81-92	1-17	94.4%	DP(truncated prenyl groups)
**Rv3806c-PRPP**	A/B/D	302/13-302		1-12	96.0%	Mg^{2+}, PRPP POPG

Supplementary Table S3: Gaussian curve fit settings and HOS structural elements designations. This table shows the peak wavenumbers of 9 Gaussians and their corresponding secondary structural motifs used to calculate the % HOS fractional contribution in Extended Data Fig. 1j.

Wavenumber	Structure motif
1626	coiled coil
1638	beta
1642	beta
1648	unordered
1656	alpha
1666	turn
1672	turn
1680	turn
1688	turn
