# Supplementary material for: Structural analysis of phosphoribosyltransferase-mediated cell wall precursor synthesis in Mycobacterium tuberculosis
Source: Nat Microbiol. 2024 Mar 15;9(4):976–87. doi: 10.1038/s41564-024-01643-8 (PMC10994848; doi:10.1038/s41564-024-01643-8)
Supplement: Supplementary file 2 — Reporting Summary [file 41564_2024_1643_MOESM2_ESM.pdf]

Corresponding author(s): Gurdyal S. Besra, Zihe Rao, and Lu Zhang

Last updated by author(s): Jan 18, 2024

Reporting Summary

Nature Portfolio wishes to improve the reproducibility of the work that we publish. This form provides structure for consistency and transparency in reporting. For further information on Nature Portfolio policies, see our Editorial Policies and the Editorial Policy Checklist.

Statistics

For all statistical analyses, confirm that the following items are present in the figure legend, table legend, main text, or Methods section.

n/a	Confirmed	
☐	☒	The exact sample size (*n*) for each experimental group/condition, given as a discrete number and unit of measurement
☐	☒	A statement on whether measurements were taken from distinct samples or whether the same sample was measured repeatedly
☒	☐	The statistical test(s) used AND whether they are one- or two-sided<br>*Only common tests should be described solely by name; describe more complex techniques in the Methods section.*
☒	☐	A description of all covariates tested
☒	☐	A description of any assumptions or corrections, such as tests of normality and adjustment for multiple comparisons
☐	☒	A full description of the statistical parameters including central tendency (e.g. means) or other basic estimates (e.g. regression coefficient) AND variation (e.g. standard deviation) or associated estimates of uncertainty (e.g. confidence intervals)
☒	☐	For null hypothesis testing, the test statistic (e.g. *F*, *t*, *r*) with confidence intervals, effect sizes, degrees of freedom and *P* value noted<br>*Give P values as exact values whenever suitable.*
☒	☐	For Bayesian analysis, information on the choice of priors and Markov chain Monte Carlo settings
☒	☐	For hierarchical and complex designs, identification of the appropriate level for tests and full reporting of outcomes
☒	☐	Estimates of effect sizes (e.g. Cohen's *d*, Pearson's *r*), indicating how they were calculated

Our web collection on statistics for biologists contains articles on many of the points above.

Software and code

Policy information about availability of computer code

Data collection	EPU2.12
Data analysis	MotionCor2 1.2.1, cryoSPRAC 4.0.1, Phenix 1.17, COOT 0.9.8, Pymol 2.1,UCSF Chimera X 1.4, GraphPad prism 9.0.

For manuscripts utilizing custom algorithms or software that are central to the research but not yet described in published literature, software must be made available to editors and reviewers. We strongly encourage code deposition in a community repository (e.g. GitHub). See the Nature Portfolio guidelines for submitting code & software for further information.

Data

Policy information about availability of data

All manuscripts must include a data availability statement. This statement should provide the following information, where applicable:

- Accession codes, unique identifiers, or web links for publicly available datasets
- A description of any restrictions on data availability
- For clinical datasets or third party data, please ensure that the statement adheres to our policy

The EM density maps generated in this study have been deposited in the EMDB under accession codes EMD-36072 (DP-bound Rv3806c), EMD-36071 (PRPP-bound Rv3806c). Atomic coordinates have been deposited in the PDB under the accession codes 8J8K (DP-bound Rv3806c), and 8J8J (PRPP-bound Rv3806c).

Research involving human participants, their data, or biological material

Policy information about studies with human participants or human data. See also policy information about sex, gender (identity/presentation), and sexual orientation and race, ethnicity and racism.

Reporting on sex and gender	not applied
Reporting on race, ethnicity, or other socially relevant groupings	not applied
Population characteristics	not applied
Recruitment	not applied
Ethics oversight	not applied

Note that full information on the approval of the study protocol must also be provided in the manuscript.

Field-specific reporting

Please select the one below that is the best fit for your research. If you are not sure, read the appropriate sections before making your selection.

☒ Life sciences ☐ Behavioural & social sciences ☐ Ecological, evolutionary & environmental sciences

For a reference copy of the document with all sections, see nature.com/documents/nr-reporting-summary-flat.pdf

Life sciences study design

All studies must disclose on these points even when the disclosure is negative.

Sample size	sample sizes were not predetermined and all available data was processed.<br>For cryo-EM, two datasets with 8894 micrographs (DP bound) and 9908 micrographs (PRPP bound) were collected and processed. Data of the DP-bound and PRPP-bound Rv3806c samples were processed to a resolution of 3.36Å and 2.76Å, both of which were sufficient for interpretation of the experimental data and to build an atomic model.
Data exclusions	No data exclusions in experimental groups except being cryo EM data processing, we discarded the "junk" particles or classes of particles that did not yield a useful 3D reconstruction. This followed the standard cryo-EM processing procedure and commonly accepted in the cryo-EM single particle analysis field.
Replication	Protein purification of WT-Rv3806c and mutant-Rv3806c, SDS-PAGE, BN-PAGE, West-Blot were repeated in at least 3 independent experiments. Scintillation counting of PRTase activity assay for all WT and mutant Rv3806c was repeated in 2-3 independent experiments. Binding affinity assays were repeated in 3-5 independent experiments.Negative stain of WT-Rv3806c and mutant-Rv3806c was repeated 3 in independent experiments. Cryo-EM sample preparation and data processing were repeated twice. The TLC-autoradiogram for the RPTase activity was performed once. Microfluidic modulation spectroscopy assay was repeated twice.All attempts at replication were successful.
Randomization	Random allocation with regard to covariate is not applicable to the experiments carried out. Experiments in this study do not necessitate randomization because they are not influenced by covariates during the sample allocation common in other research methods.
Blinding	Blinding was not required for this study because nor subjective allocation was involved, neither did data processing rely on subjective assignments. Because the biochemical/biophysical data was visualized by scintillation counting, or absorbance and quantified using standard software, which did not require subjective analysis. And structural data analyzed by standard software packages which didnot require subjective judgment.

Reporting for specific materials, systems and methods

We require information from authors about some types of materials, experimental systems and methods used in many studies. Here, indicate whether each material, system or method listed is relevant to your study. If you are not sure if a list item applies to your research, read the appropriate section before selecting a response.

Materials & experimental systems

n/a	Involved in the study	
☐	☒	Antibodies
☒	☐	Eukaryotic cell lines
☒	☐	Palaeontology and archaeology
☒	☐	Animals and other organisms
☒	☐	Clinical data
☒	☐	Dual use research of concern
☒	☐	Plants

Methods

n/a	Involved in the study	
☒	☐	ChIP-seq
☒	☐	Flow cytometry
☒	☐	MRI-based neuroimaging

Antibodies

Antibodies used	HPR-conjugated mouse anti DDDDK-Tag(1:5000,ABClone,AE024,China) was used for the Western Blot analysis of WT and mutant Rv3806c.
Validation	Antibody was exclusively used for western blotting. Validation of the antibody can be found on the website (https://abclonal.com.cn/catalog/AE024).
