# Supplementary figures and images for: Structural analysis of phosphoribosyltransferase-mediated cell wall precursor synthesis in Mycobacterium tuberculosis
Source: Nat Microbiol. 2024 Mar 15;9(4):976–87. doi: 10.1038/s41564-024-01643-8 (PMC10994848; doi:10.1038/s41564-024-01643-8)

# Sample: Buffer

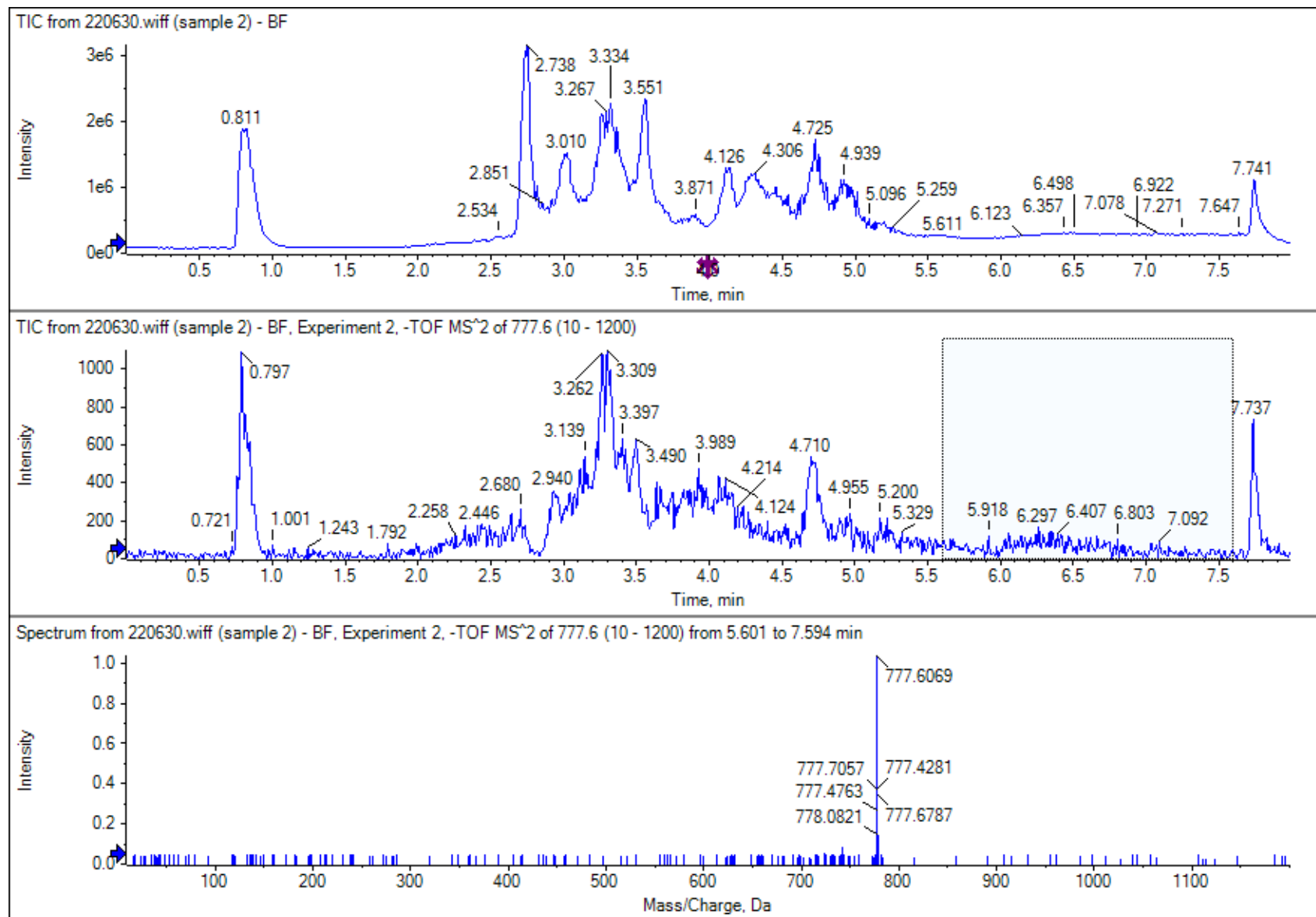

Sample: 0.04mg/ml Rv3806c

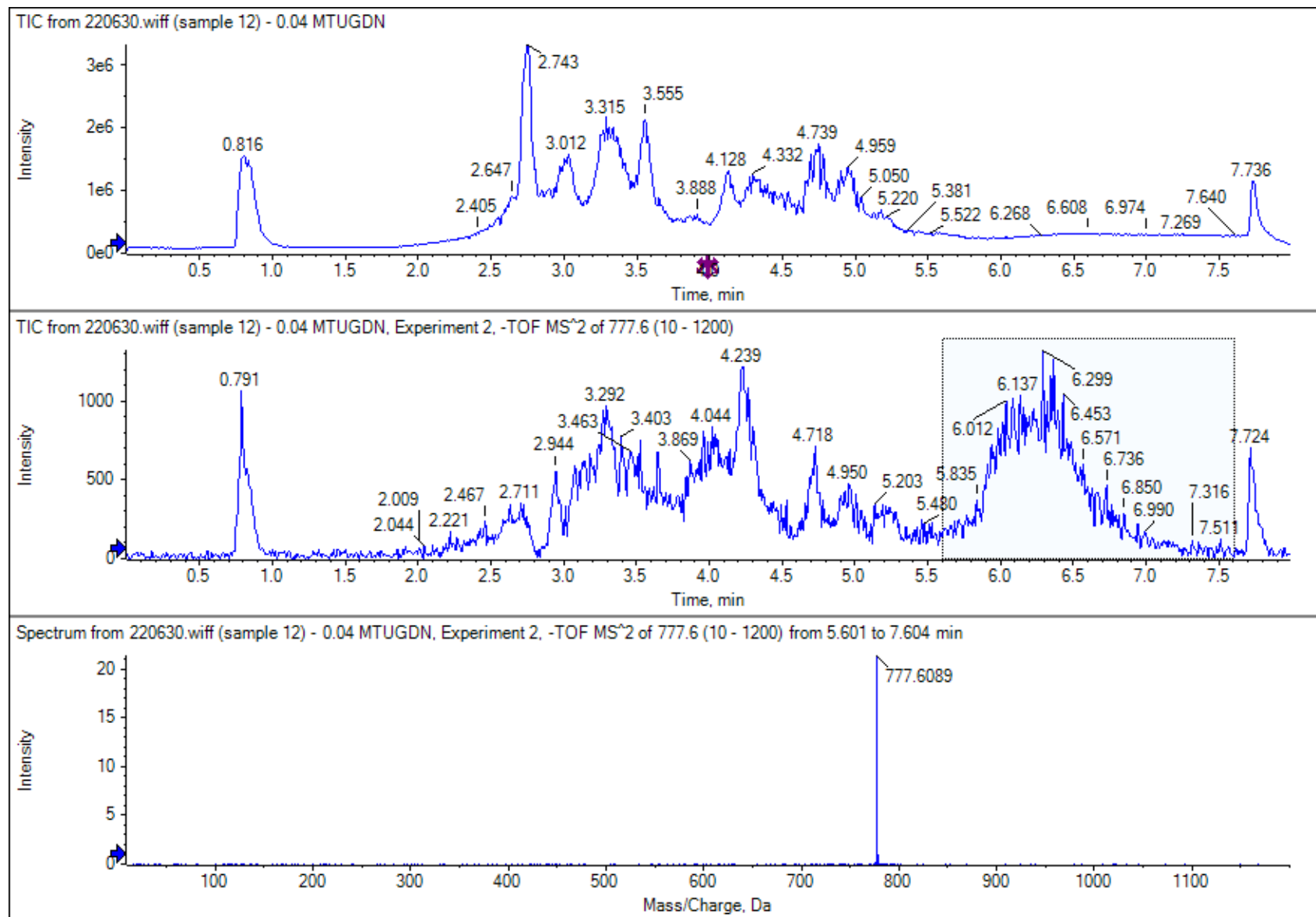

# Sample: 0.2mg/ml Rv3806c

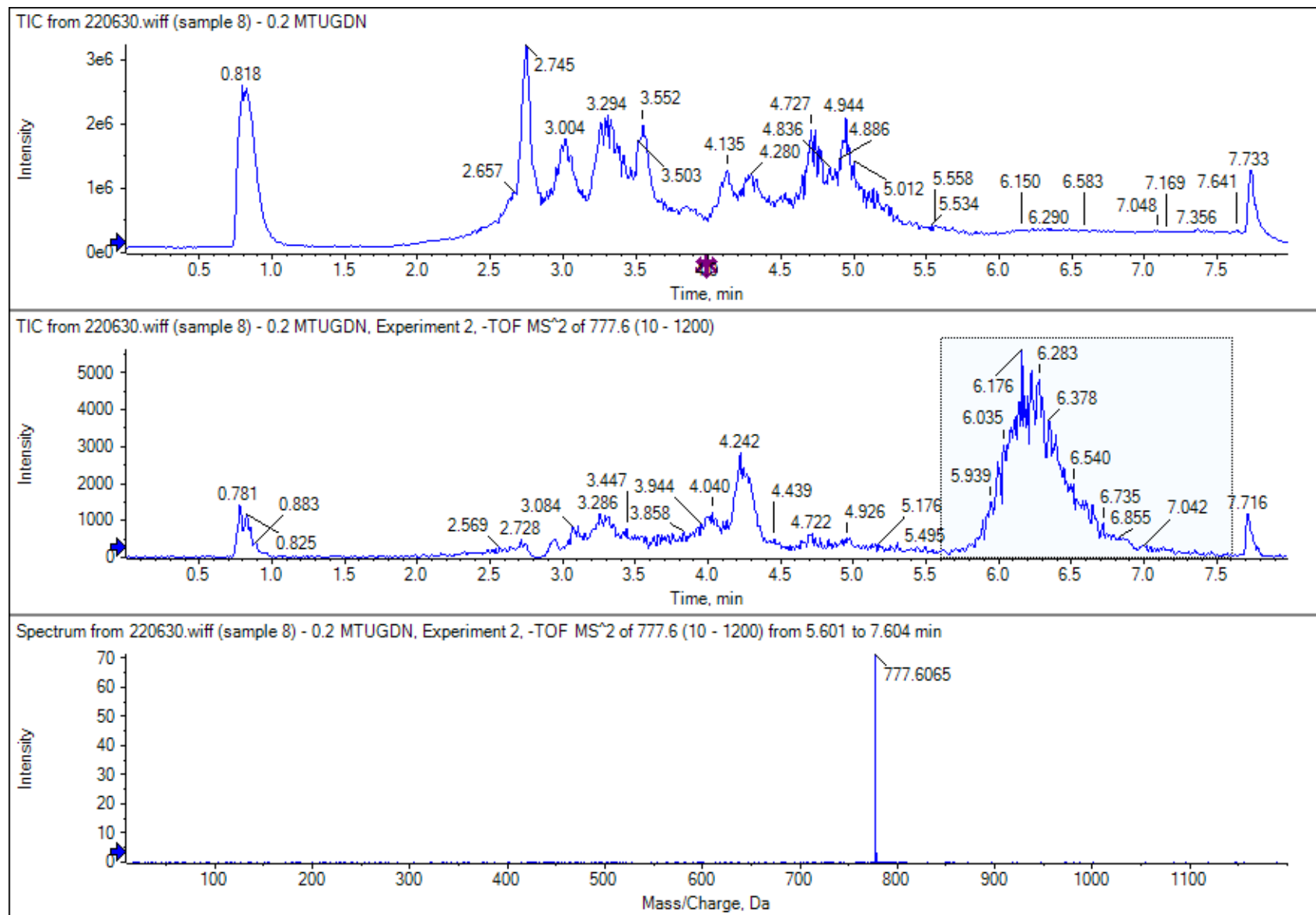

# Sample: 1.0mg/ml Rv3806c

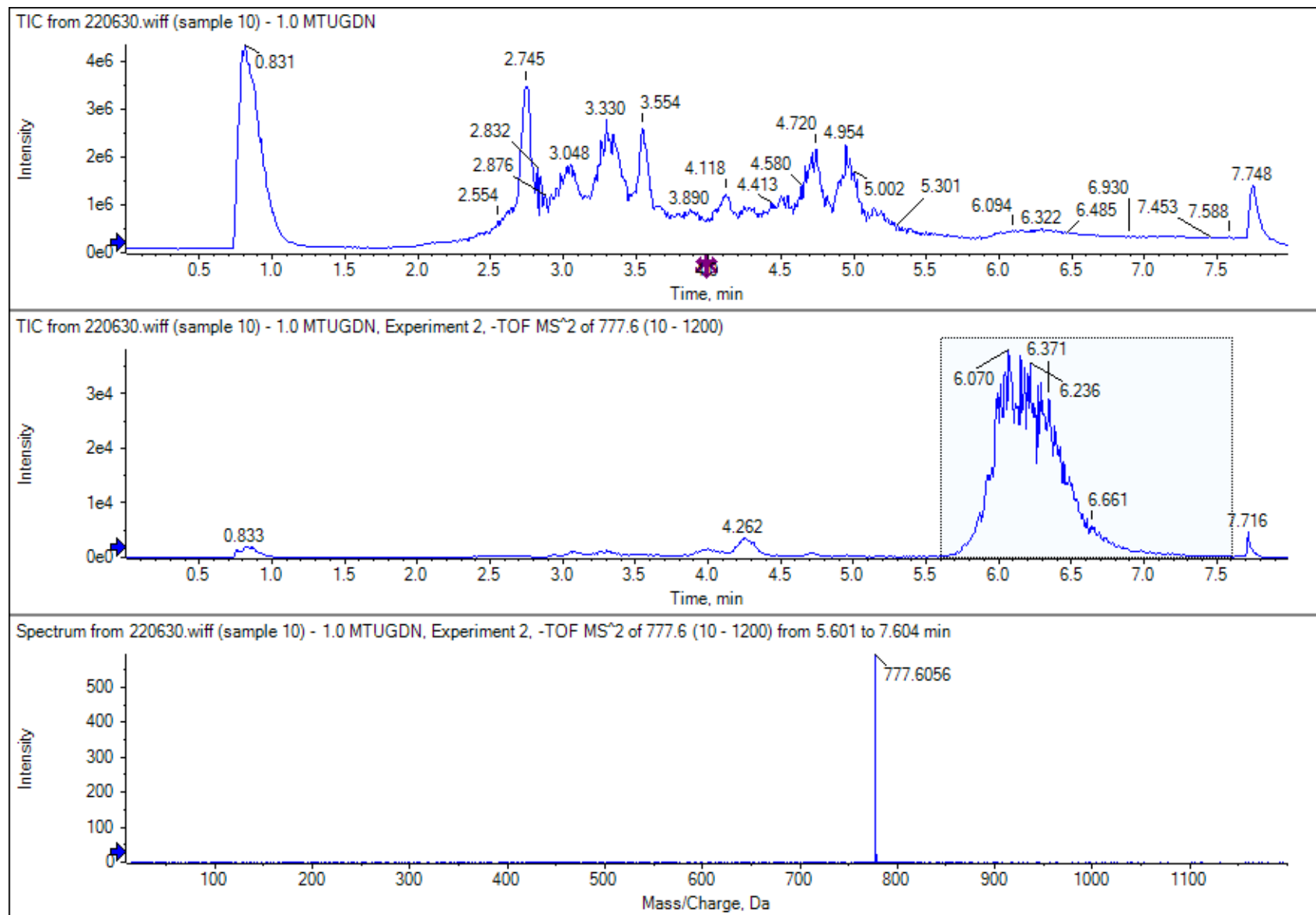

Supplement: Supplementary file 9 — Unprocessed SDS–PAGE for Extended Data Fig. 1b. Unprocessed TLC autoradiogram for Extended Data Fig. 1c. Unprocessed SDS–PAGE for Extended Data Fig. 1e. Unprocessed western blot for Extended Data Fig. 1g. Unprocessed native PAGE for Extended Data Fig. 1h. Unprocessed statistical source data for Extended Data Fig. 1k. Unprocessed software raw data for Extended Data Fig. 1k. [file 41564_2024_1643_MOESM9_ESM.zip › Gao_SourceData_Extended_Data_Fig1/Extended_Data_Fig1k_Mass_spectrometry.pdf]

Red rectangles indicate the areas that are displayed in the paper

**Extended Data Fig. 1c**

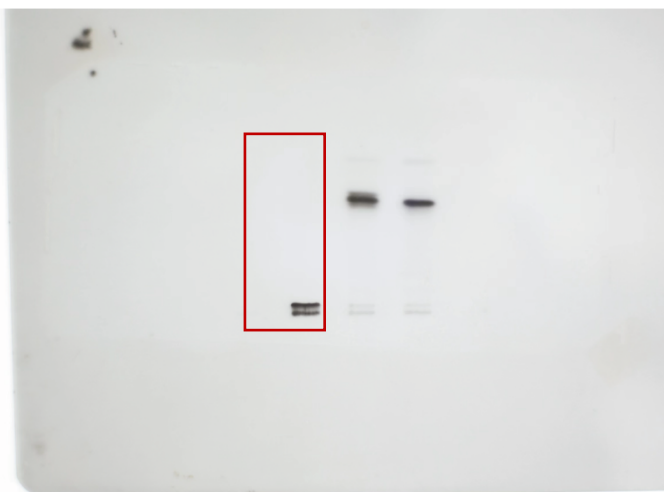

Supplement: Supplementary file 9 — Unprocessed SDS–PAGE for Extended Data Fig. 1b. Unprocessed TLC autoradiogram for Extended Data Fig. 1c. Unprocessed SDS–PAGE for Extended Data Fig. 1e. Unprocessed western blot for Extended Data Fig. 1g. Unprocessed native PAGE for Extended Data Fig. 1h. Unprocessed statistical source data for Extended Data Fig. 1k. Unprocessed software raw data for Extended Data Fig. 1k. [file 41564_2024_1643_MOESM9_ESM.zip › Gao_SourceData_Extended_Data_Fig1/Extended_Data_Fig1c_TLC-autoradiogram.pdf]

Red rectangles indicate the areas that are displayed in the paper

**Extended Data Fig. 1e**

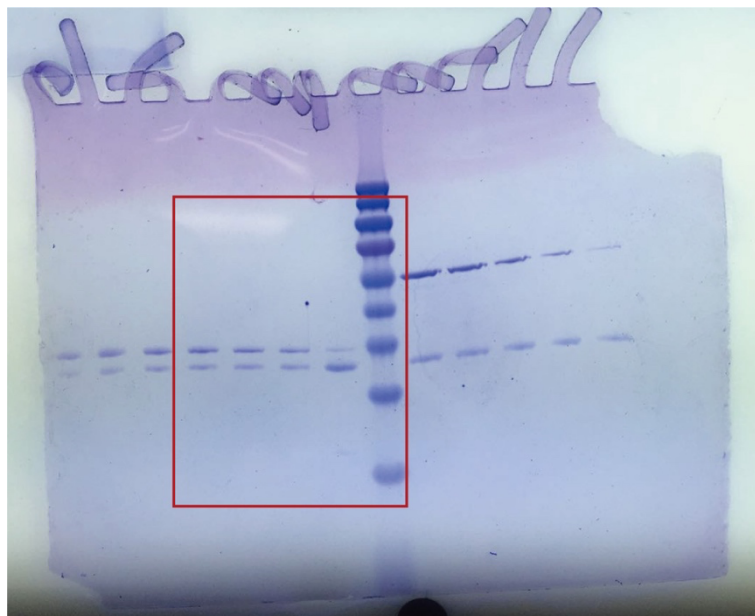

Supplement: Supplementary file 9 — Unprocessed SDS–PAGE for Extended Data Fig. 1b. Unprocessed TLC autoradiogram for Extended Data Fig. 1c. Unprocessed SDS–PAGE for Extended Data Fig. 1e. Unprocessed western blot for Extended Data Fig. 1g. Unprocessed native PAGE for Extended Data Fig. 1h. Unprocessed statistical source data for Extended Data Fig. 1k. Unprocessed software raw data for Extended Data Fig. 1k. [file 41564_2024_1643_MOESM9_ESM.zip › Gao_SourceData_Extended_Data_Fig1/Extended_Data_Fig1e_SDSΓÇôPAGE.pdf]

Red rectangles indicate the areas that are displayed in the paper

**Extended Data Fig. 1b**

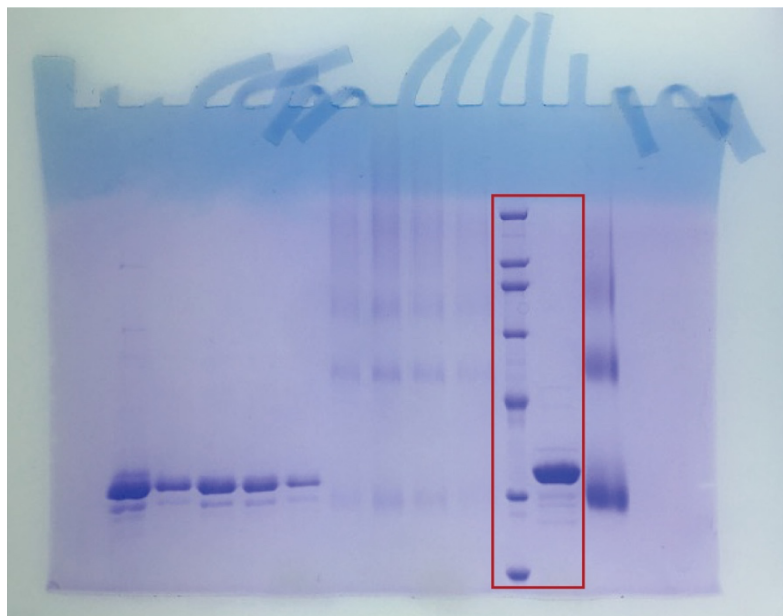

Supplement: Supplementary file 9 — Unprocessed SDS–PAGE for Extended Data Fig. 1b. Unprocessed TLC autoradiogram for Extended Data Fig. 1c. Unprocessed SDS–PAGE for Extended Data Fig. 1e. Unprocessed western blot for Extended Data Fig. 1g. Unprocessed native PAGE for Extended Data Fig. 1h. Unprocessed statistical source data for Extended Data Fig. 1k. Unprocessed software raw data for Extended Data Fig. 1k. [file 41564_2024_1643_MOESM9_ESM.zip › Gao_SourceData_Extended_Data_Fig1/Extended_Data_Fig1b_SDSΓÇôPAGE.pdf]

Red rectangles indicate the areas that are displayed in the paper

**Extended Data Fig. 1g**

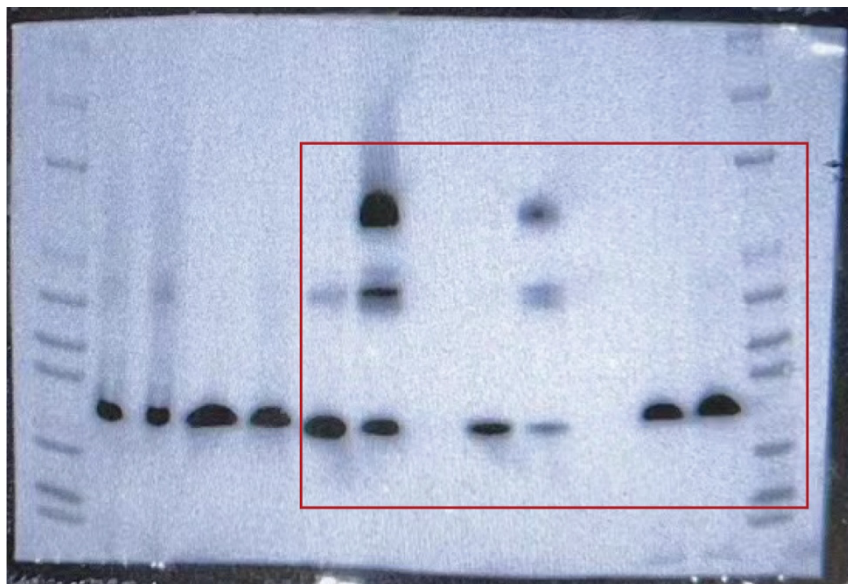

Supplement: Supplementary file 9 — Unprocessed SDS–PAGE for Extended Data Fig. 1b. Unprocessed TLC autoradiogram for Extended Data Fig. 1c. Unprocessed SDS–PAGE for Extended Data Fig. 1e. Unprocessed western blot for Extended Data Fig. 1g. Unprocessed native PAGE for Extended Data Fig. 1h. Unprocessed statistical source data for Extended Data Fig. 1k. Unprocessed software raw data for Extended Data Fig. 1k. [file 41564_2024_1643_MOESM9_ESM.zip › Gao_SourceData_Extended_Data_Fig1/Extended_Data_Fig1g_Western Blot.pdf]

Red rectangles indicate the areas that are displayed in the paper

**Extended Data Fig. 1h**

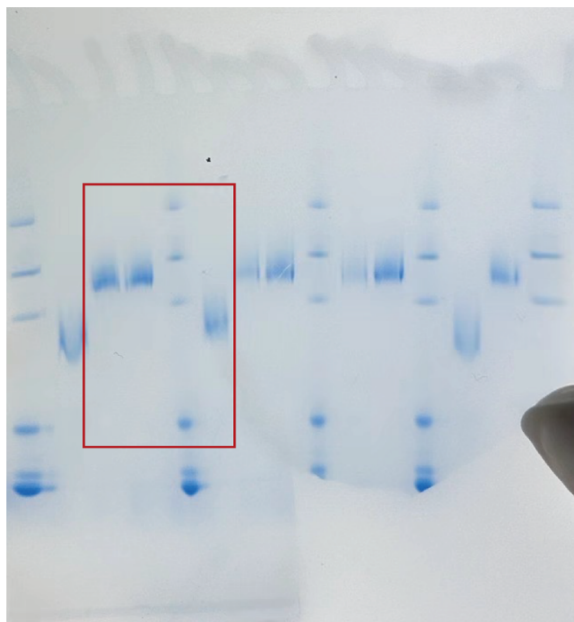

Supplement: Supplementary file 9 — Unprocessed SDS–PAGE for Extended Data Fig. 1b. Unprocessed TLC autoradiogram for Extended Data Fig. 1c. Unprocessed SDS–PAGE for Extended Data Fig. 1e. Unprocessed western blot for Extended Data Fig. 1g. Unprocessed native PAGE for Extended Data Fig. 1h. Unprocessed statistical source data for Extended Data Fig. 1k. Unprocessed software raw data for Extended Data Fig. 1k. [file 41564_2024_1643_MOESM9_ESM.zip › Gao_SourceData_Extended_Data_Fig1/Extended_Data_Fig1h_NativeΓÇôPAGE.pdf]
